# Understanding patient experiences: The powerful source of written patient stories

**DOI:** 10.1111/hex.13053

**Published:** 2020-04-01

**Authors:** Hester M. van de Bovenkamp, Coleta Platenkamp, Roland Bal

**Affiliations:** ^1^ Erasmus School of Health Policy & Management Erasmus University Rotterdam Rotterdam The Netherlands; ^2^ Founder of Foundation Coleta's Chronic Circus Rotterdam The Netherlands

Telling stories of illness is the attempt, instigated by the body's disease, to give a voice to an experience that medicine cannot describe. (Frank, 2013, p. 18)

The value and benefits of patient stories are increasingly recognized. A recent editorial in this journal emphasized the importance of these stories for improving the quality of health‐care services.^1^ Similarly, using patient stories for self‐help and research are propagated.^2^, ^3^ Placing patient stories centre stage is felt to be important as stories offer much more in‐depth information compared to, for example, satisfaction questionnaires and thereby allowing for a better understanding of patient experiences with treatment, health‐care services and daily life with a condition.^4^ This increased attention for patient stories has resulted in the use of many story collecting methods such as interviews, focus groups, mirror meetings and experience‐based co‐design. Strangely, a readily available source, written patient stories, is overlooked in much of the literature. In this contribution, we focus on the added value of these written patient stories for peer support, patient representation, quality improvement, policymaking, education and research.^5^ Studying stories can strengthen our knowledge base and contribute to the development of Patient Sciences, focusing on the study of patient experiences, patient participation and representation.^5^

There are many patients who put their stories into writing. In the Netherlands alone, more than 300 are published each year. The foundation Coleta's Chronic Circus has collected thousands of these stories throughout the years and described them on its website.^*^
https://patientervaringsverhalen.nl/
 These stories are now part of the collection of Erasmus University Rotterdam, making them available to a wider (Dutch reading) audience.^†^
https://www.eur.nl/library/collecties/collectie‐patientervaringen



An important advantage of these written stories is that they offer insight into all interrelated facets of life with a condition. This includes ‘objective’ medical complaints, social aspects and the experience of being sick.^6^ Written stories provide insight into the experiences of patients in everyday life without being pre‐structured by other actors, such as quality improvement workers, policymakers or researchers. For example, when conducting an evaluation of a government‐funded programme to tackle ‘disoriented behaviour’ (which appeared prominently on the Dutch policy agenda after several violent crimes committed by people with mental health problems), a review of the stories from those that possibly fit into this category allowed us to better understand the impact of their condition on all aspects of their lives. This includes how they frame the problems they encounter as well as being subjected to ‘disoriented behaviour’ discourse.^7^

Additionally, written stories provide insight on how patient perspectives can change during their life, thereby providing internal diversity. For instance, the perspective from parents of children with Down syndrome can change over time from being a medical condition to (also) being part of their child's identity. These insights are informative and have consequences on how professionals should approach parents and their child but also for broader policy discussions such as prenatal testing and the type of information that should be provided to future parents regarding the choices surrounding such tests.^8^

Written stories also offer external diversity as analysing different stories provides us with the opportunity to consider the differences between patients and between patients and their loved ones (who write many stories as well). For example, there are many stories written by mothers that have a child with a condition. This offers insight into a variety of maternal experiences that exhibit similarities (eg feeling helpless and exhausted from being a care manager all the time) and differences (eg their children's needs, finances, jobs, family situation, religion and professionals they encounter). An analysis of these stories can be complemented with an analysis of stories from the children themselves as adults. This offers a unique opportunity to study how their perspective might differ from their mothers’.^‡^See for example, http://www.annemariehaverkamp.nl/job/, https://www.patientervaringsverhalen.nl/images/abook_file/605.pdf, https://www.patientervaringsverhalen.nl/?id=30276, https://www.facebook.com/EbelsDroom/posts/welkom‐bij‐de‐pagina‐van‐ebels‐droom‐we‐hopen‐dat‐het‐bovenstaande‐verhaal‐zie‐a/598846770180041
 Similarly, comparisons can be made between the stories of patients suffering from different conditions in order to identify themes that are specific to a particular condition and those that are more general, such as fatigue or the temporality of chronic living. Moreover, written stories allow for a historical analysis of patient experiences.

Analysing written patient stories has many advantages as they allow us to gain a broad and in‐depth understanding of patient experiences. Other types of stories, such as documentaries and vlogs, can be of further value as they focus on specific situations that are of special importance to patients or help to gain insight into the experiences of young patients who more frequently use this medium.^9^ Other sources used to collect patient stories can also be of value. Interviews and observations can provide more information on specific issues^10^, ^11^ and be a means to collect stories from patients who are unable to write their stories, for example because their life with their condition is too ‘chaotic’ or ‘broken’.^6^

Written stories and the analysis thereof can be of interest to a wide variety of actors. Patients and their loved ones can use them to draw lessons about (living with) their condition, whereas professionals can use them to develop a better understanding of patient experiences and patient knowledge and provide more patient‐centred care. Patient organizations and other actors making representative claims on behalf of patients^12^ can strengthen their knowledge and activities by utilizing these stories in their representation efforts. These stories can also enable policymakers to create a better fit between their policies and patient experiences. Teachers active in medical and health‐care management education can include these stories in their educational activities. Experiences in our own teaching practices demonstrate that students very much value learning about patient perspectives in this way. One of them explained that for him it brought back the emotional experience that is essential in health care, which he often felt to be lacking in scientific literature and policy reports. Researchers can study this unique source to gain a much better understanding of patient knowledge and experiences and translate their findings to the aforementioned stakeholders.

To conclude, with this contribution we would like to draw attention to a readily available but often overlooked valuable source of information regarding patient experiences, those of written patient stories. These stories can offer a crucial contribution to research on patient experiences, improve the quality of care for patients and improve health‐care systems based on these experiences.
